# Development and validation of a real-time PCR assay for detection and quantification of *Tuber magnatum* in soil

**DOI:** 10.1186/1471-2180-12-93

**Published:** 2012-06-06

**Authors:** Mirco Iotti, Marco Leonardi, Marilena Oddis, Elena Salerni, Elena Baraldi, Alessandra Zambonelli

**Affiliations:** 1 Dipartimento di Protezione e Valorizzazione Agroalimentare, Alma Mater Studiorum Università di Bologna, via Fanin 46, 40127, Bologna, Italy; 2 Dipartimento di Scienze Ambientali, Università dell’Aquila, via Vetoio, Coppito 1, 67100, L’Aquila, Italy; 3 Dipartimento di Scienze Ambientali “G. Sarfatti”, Università degli Studi di Siena, via Mattioli 4, 53100, Siena, Italy

**Keywords:** Real-time PCR, Taq-man probe, *Tuber magnatum* DNA concentration, Soil DNA extraction, ITS primers, Truffle production

## Abstract

**Background:**

*Tuber magnatum*, the Italian white truffle, is the most sought-after edible ectomycorrhizal mushroom. Previous studies report the difficulties of detecting its mycorrhizas and the widespread presence of its mycelium in natural production areas, suggesting that the soil mycelium could be a good indicator to evaluate its presence in the soil. In this study a specific real-time PCR assay using TaqMan chemistry was developed to detect and quantify *T. magnatum* in soil. This technique was then applied to four natural *T. magnatum* truffières located in different regions of Italy to validate the method under different environmental conditions.

**Results:**

The primer/probe sets for the detection and quantification of *T. magnatum* were selected from the ITS rDNA regions. Their specificity was tested *in silico* and using qualitative PCR on DNA extracted from 25 different fungal species. The *T. magnatum* DNA concentration was different in the four experimental truffières and higher in the productive plots. *T. magnatum* mycelium was however also detected in most of the non-productive plots. Ascoma production during the three years of the study was correlated with the concentration of *T. magnatum* DNA.

**Conclusions:**

Taken together, these results suggest that the specific real-time PCR assay perfected in this study could be an useful tool to evaluate the presence and dynamics of this precious truffle in natural and cultivated truffières.

## Background

Truffles are hypogeous ectomycorrhizal Ascomycetes belonging to the order Pezizales. The most sought-after species belong to the *Tuber* genus and include *Tuber melanosporum* Vittad. (Périgord black truffle), *Tuber magnatum* Pico (Italian white truffle), *Tuber aestivum* Vittad. (Burgundy truffle) and *Tuber borchii* Vittad. (bianchetto). Amongst these the Italian white truffle commands the highest prices. This truffle grows in many regions of Italy: from Piedmont in the north, where Alba is the most famous production area, to Basilicata in the extreme south of Italy [1]. It is also found in Croatia and has recently been found, although in small quantities, in Romania, Serbia, Hungary and Slovenia [2-4].

Methods have been developed to produce *T. magnatum* infected trees using spore inoculation techniques [5-7] or root organ cultures [8]. However, while some successes are reported [9] in general attempts to cultivate this truffle species have met with failure [1,10,11]. This failure to produce *T. magnatum* fruiting bodies from cultivated plots has been compounded by falling harvests from natural truffières, attributed to deforestation, changing forest management practices, global warming since the last ice age as well as acid rain [12]. These factors have spurred efforts to carry out research aimed at safeguarding *T. magnatum* production in natural truffières and developing tools to evaluate their state of “health”.

In contrast to the other truffles such as *T. melanosporumT. aestivum* and *T. borchii*, which are comparatively easy to cultivate, *T. magnatum* mycorrhizas are scarce or absent even where their ascomata are found [13,14]. On the other hand, recent studies have shown that *T. magnatum* mycelium is widely distributed in the soil of truffières and its presence is not restricted to just those points where mycorrhizas or ascomata are found [15]. These observations suggest that *T. magnatum* soil mycelium could be a better indicator than mycorrhiza for assessing its presence in the soil.

DNA-based techniques have been extensively applied to study fungal ecology in soil [16]. Recently, real-time PCR has made it possible not only to detect and monitor the distribution of a particular fungus but also its abundance [17-20]. Knowledge of the distribution, dynamics and activities of *Tuber* spp. mycelium in soil can be considered crucial for monitoring the status of a cultivated truffle orchard before ascoma production [21]. It is also a powerful tool for assessing truffle presence in natural forests in those countries where ascoma harvesting is forbidden [22] or where all truffle collectors have open access to forests and woodlands [1]. This is particularly important for *T. magnatum* as the truffle production sites, in natural truffières, are dispersed and not visible to the naked eye, unlike black truffles (*T. melanosporum* and *T. aestivum*) which produce burnt areas (called “brûlée” in France, “bruciate” or “pianello” in Italy) around the productive trees where grass development is inhibited [1].

In this study a specific real-time PCR assay using TaqMan chemistry was developed to detect and quantify *T. magnatum* in soil. This technique was then applied to four natural *T. magnatum* truffières in different Italian regions to validate the method under different environmental conditions.

## Results and discussion

### DNA extraction

Successful application of molecular-based techniques for DNA analyses of environmental samples strongly depends on the quality of the DNA extracted [23]. Moreover, the heterogeneous distribution of fungi in soil with small samples (<1 g) can lead to an unrepresentative fungal fingerprinting [24]. For this reason total DNA was isolated from 15 g of lyophilized soil for each plot (3 sub-samples of 5 g each), selected from about 60 g of sampled soil from each plot, using a procedure specifically developed to obtain good quality extracts regardless of the different soil types analysed in this study. To obtain equal 3 ml-solutions of crude DNA from the different soils we had to process samples from Emilia-Romagna/Tuscany and Molise/Abruzzo truffle areas with different quantities of CTAB lysis buffer (6 and 7 ml respectively) at the beginning of the extraction step. A total of 351 extractions (3 replicates per 117 soil samples) were successfully carried out using this improved method. The mean quantity of DNA isolated from samples processed in this study range from 2.2 to 7.0 μg g^−1^ of soil for the Molise and Tuscan truffières respectively.

ANOVA was performed to determine whether the quantities of DNA isolated from the sampled soil varied in the different truffières. The data reveal significant differences (*p* ≤ 0.05) between DNA isolated from the soil samples of the different truffières (Table 1). The lowest values were obtained from samples collected in the Molise and Abruzzo truffières. This may be due to the higher clay content in the soil of these two experimental truffières. Indeed, DNA extraction is difficult for soils containing clay [25,26] and DNA adsorption and desorption is strongly affected by the clay type and content [27]. Other factors such as climate, soil, and vegetation conditions may however also contribute to modifying microbial activity below ground and consequently the quantity of total DNA isolated.

**Table 1:** Mean values and statistics of soil DNA extractions and real time PCRs

**Truffière locality (region)**	**Soil DNA extraction**^**1**^			**PP/TNP**	**Real time data**^**1**^			
	**quantity (μg g**^**-1**^** soil)**^**2**^	**OD**_**260/230 nm**_	**OD**_**260/280 nm**_		**plot with TM-DNA/TNP**	**TM-DNA concentration**^**3**^		
						**Whole**^**2**^	**PP**	**NPP**^**4**^
Feudozzo (A)	3.4 a	1.75	1.79	6/12	12/12	8.46 a	9.85	7.08
Collemeluccio (M)	2.3 a	1.64	1.64	1/9	5/9	0.72 a	3.12	0.03*
Argenta (ER)	6.9 b	1.81	1.83	4/9	8/9	11.76 a	19.28	5.73*
Barbialla (T)	7.0 b	1.82	1.83	6/9	9/9	28.18 b	35.41	13.71

^1^Mean values referred to three years of experimentation.

^2^Different letters in the same column indicate significant differences between the mean values obtained from different truffières (ANOVA and Bonferroni’s test, *p* < 0.05).

^3^pg of *T. magnatum* DNA in 200 ng of total DNA.

^4^ The asterisk indicates significant differences between the mean TM-DNA concentration of PP and NPP in the same truffière (ANOVA, *p* < 0.05).

A, Abruzzo; M, Molise; ER, Emilia Romagna; T, Tuscany; OD, optical density; PP, productive plots; NPP, non productive plots; TNP, total number of plots; TM-DNA, *T. magnatum* DNA.

Mean values of the OD_260/280 nm_ and OD_260/230 nm_ ratios calculated for each truffière range from 1.73 to 1.77 and from 1.65 to 1.71 respectively.

### Primer and probe selection

The ITS regions were chosen to develop an appropriate primer/probe set for the detection and quantification of *T. magnatum*. The use of these genomic regions as the target for real time PCR-amplification has proven to be a successful strategy for different ectomycorrhizal fungi in soil [19,21,28]. This is due to the large number of sequences available in genetic databases that make ITS regions suitable for designing reliable species-specific primers. Moreover, the presence of multiple copies of rDNA units within each fungal genome also make it possible to detect low quantities of the target DNA [29]. ITS regions are not, however, equally variable in all groups of fungi [30] and this could represent a limitation for designing a specific primer in some species [31]. The alignment of about 70 ITS1-5.8 S-ITS2 *T. magnatum* sequences retrieved from the GenBank database highlighted a high level of conservation of ITS regions in this species (0/186 nt for ITS1 and 2/217 for ITS2), higher than those found in other truffle species [32-34].

**Figure 1:**
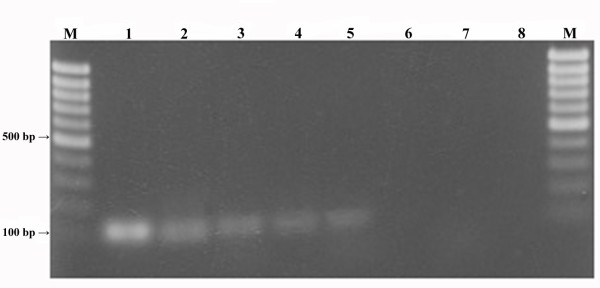
**PCR sensitivity of the primer pairs selected from ITS1 and ITS2 regions.** Reactions carried out using serial dilutions of *T. magnatum* DNA (TM-DNA) in pooled non-target fungal DNAs (F-DNA): lane M, Mass ruler marker (Fermenats); lanes 1, 3, 5 and 7, ITS1for-ITS1rev primer pair; lanes 2, 4, 6 and 8, ITS2for-ITS2rev primer pair. Lanes 1–2, 10 ng TM-DNA/90 ng F-DNA; lanes 3–4, 1 ng TM-DNA/99 ng F-DNA; lanes 5–6, 0.1 ng TM-DNA/99.9 ng F-DNA; lanes 7–8, 0.01 ng TM-DNA/99.99 ng F-DNA.

A single primer/probe set was selected for both the ITS1 and the ITS2 region (Table 2) based on *in silico* analyses of their composition, Tm, PCR-impairing structure formation and specificity against the sequences in GenBank. Both of the primer pairs selected produced specific amplicons of the expected size for all the *T. magnatum* specimens considered in this study and gave no cross-reactions with other fungal species under qualitative PCR conditions (Table 3). Specificity of the probes was also confirmed (data not shown). However, the primers and probe designed from ITS1 were selected for the subsequent real-time PCR analyses, as they provided more efficient amplification (Figure 1). Indeed, the TmgITS1for-TmgITS1rev primer pair allowed detection of the specific amplicon down to dilutions of 1/1000 (0.1 ng of *T. magnatum* DNA mixed with 100 ng of non-target DNAs), ten fold lower than TmgITS2for-TmgITS2rev. The specificity of the ITS1 primer/probe set was also confirmed under real-time PCR conditions for all soil samples processed.

**Table 2:** Primers and probes tested in this study

**Primer/Probe**	**Sequence (5′-3′)**	**Length (bp)**	**Amplicon (bp)**	**Target region**	**GC (%)**
TmgITS1for	GCGTCTCCGAATCCTGAATA	20	106	ITS1	50
TmgITS1rev	ACAGTAGTTTTTGGGACTGTGC	22			45
TmgITS1prob	TGTACCATGCCATGTTGCTT	20			45
TmgITS2for	AAACCCACTCACGGAATCAC	20	99	ITS2	50
TmgITS2rev	CGTCATCCTCCCAATGAAA	19			47
TmgITS2prob	GTACCAAGCCACCTCCATCA	20			55

**Table 3:** Collection numbers and origin of the fungal materials used in this study

**Species**	**Source**^**1**^	**CMI-Unibo**^**2**^**herbarium code**	**Origin (Region, Country)**
*Tuber magnatum* Pico	d.A	CMI-Unibo 1182	Molise, Italy
*Tuber magnatum* Pico	d.A	CMI-Unibo 3990	Emilia Romagna, Italy
*Tuber magnatum* Pico	d.A	CMI-Unibo 4059	Marche, Italy
*Tuber magnatum* Pico	d.A	CMI-Unibo 4090	Romania
*Tuber magnatum* Pico	d.A	CMI-Unibo 4152	Emilia Romagna, Italy
*Tuber aestivum* Vittad.	d.A	CMI-Unibo 1571	Marche, Italy
*Tuber asa* Tul. & C. Tul.	d.A	CMI-Unibo 2124	Veneto, Italy
*Tuber borchii* Vittad. (type 1)^3^	d.A	CMI-Unibo 2682	Sicily, Italy
*Tuber borchii* Vittad. (type 2)^3^	d.A	CMI-Unibo 2363	Veneto, Italy
*Tuber brumale* Vittad.	d.A	CMI-Unibo 1547	Emilia Romagna, Italy
*Tuber dryophilum* Tul. & C. Tul.	d.A	CMI-Unibo 1547	Emilia Romagna, Italy
*Tuber excavatum* Vittad.	d.A	CMI-Unibo 1446	Emilia Romagna, Italy
*Tuber indicum* Cooke and Massee	d.A	CMI-Unibo 1759	Yunnan, China
*Tuber macrosporum* Vittad.	d.A	CMI-Unibo 1515	Emilia Romagna, Italy
*Tuber maculatum* Vittad.	M	Tma1	Emilia Romagna, Italy
*Tuber melanosporum* Vittad.	M	Tme4	Marche, Italy
*Tuber mesentericum* Vittad.	d.A	CMI-Unibo 1585	Emilia Romagna, Italy
*Tuber oligospermum* (Tul. & C. Tul.) Trappe	d.A	CMI-Unibo 4231	Marmora forest, Morocco
*Tuber rufum* Pico	d.A	CMI-Unibo 1798	Emilia Romagna, Italy
*Terfezia claveryi* Chatin	d.A	CMI-Unibo 4231	Cappadocia, Turkey
*Choiromyces meandriformis* Vittad.	d.A	CMI-Unibo 1432	Emilia Romagna, Italy
*Balsamia vulgaris* Vittad.	d.A	CMI-Unibo 3460	Emilia Romagna, Italy
*Genea klotzschii* Berk. & Broome	d.A	CMI-Unibo 1944	Emilia Romagna, Italy
*Ganoderma lucidum* (Curtis) P. Karst.	M	Glu5039	Armenia
*Hymenogaster luteus* Vittad.	d.B	CMI-Unibo 1947	Emilia Romagna, Italy
*Valsa ceratosperma* (Tode) Maire	M	Vce155	Emilia Romagna, Italy
*Cryphonectria parasitica* (Murrill) M.E. Barr.	M	Cpa5	Emilia Romagna, Italy
*Monilia laxa* (Ehrenb.) Sacc. & Voglino	M	Mla95	Emilia Romagna, Italy
*Aspergillus flavus* Link	M	Afl7	Emilia Romagna, Italy
*Penicillium expansum* Link	M	Pex25	Emilia Romagna, Italy

^1^ d.A = dried ascoma; d.B = dried basidioma; M = mycelium in pure culture.

^2^ CMI-Unibo **=** Center of mycology of Bologna University.

^3^ Bonuso et al. [35].

### Real time quantification of *T. magnatum* DNA

**Figure 2:**
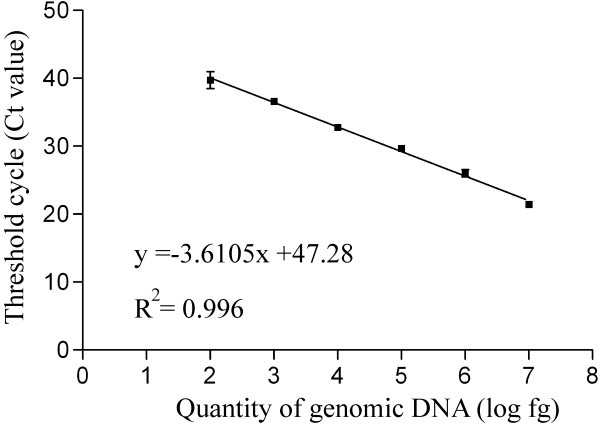
**Real-time PCR standard curve for*T. magnatum*DNA quantification.** The curve was generated by plotting the means of the Ct values obtained against the logarithm of a known quantity of genomic DNA. Variability is shown as the mean Ct value ± SD.

The real-time assay showed reliable amplification over the 6 orders of magnitude generating almost identical standard curves from each run quantifying *T. magnatum* DNA in soil samples. The correlation coefficients (R^2^ values) were always higher than 0.99 and amplification efficiency was about 85%. The mean standard curve resulting from 18 independent plates is shown in Figure 2. The detection limit for real-time PCR with the ITS1 primer/probe set was approximately 10 fg. However, since standard replicates containing less than 100 fg of *T. magnatum* DNA gave inconsistent amplifications, to avoid the inclusion of false positive test results, values lower than this threshold were considered as 0.

### Detection of *T. magnatum* ascomata and DNA

Truffle production was scattered and localized in only 17 of the 39 plots examined. A total of 74 *T. magnatum* ascomata, for a total weight of 1184.3 g, were collected over the 3-year period of investigation in the 4 experimental truffières (Additional file 1).

There was a high variation in the concentration of *T. magnatum* DNA detected by real-time PCR in the 117 samples processed, even from the same plot, over the three years of sampling thus confirming that mycelium varies considerably in the soil over time [28]. No fluorescence was ever recorded in DNA from the soil samples collected outside the truffière in any of the experimental sites.

The mean concentration of *T. magnatum* DNA detected in the four different truffières was statistically different indicating that environmental condition, such as climate, vegetation, soil chemical and biological characteristics, influence the relative quantity of *T. magnatum* DNA in the soil (Table 1). The lowest mean concentration of target DNA was associated with the soil samples collected in the Molise truffière. In this experimental site significant amounts of *T. magnatum* DNA were only detected in the unique plot that produced ascomata during the 3 years of the survey. On the contrary, soil samples from the Tuscan truffière showed the highest mean value for DNA concentration and positive real-time amplifications were obtained for all plots. *T. magnatum* DNA was also found in plots that never produced truffles during the three years of the study (Table 1). This can be explained by the fact that, in soil, *T. magnatum* mycelium is able to develop as far as 100 m from the production points [15], thus forming large mycelial patches that may colonize other contiguous plots. Higher mean values for *T. magnatum* DNA concentrations were however obtained from productive plots (Table 1) even if in Tuscany and Abruzzo no significant differences were found between productive and non-productive plots. This is probably due to the high percentage of productive plots of these two truffières where mycelial patches may have overlapped. Despite this, there was a significant correlation (p-level ≤ 0.05) between the mean *T. magnatum* DNA concentration and plot productivity (Spearman's rank correlation coefficients, respectively 0.56 and 0.55 for the number and the weight of ascomata collected in the three years of the study). These results indicate that the production of *T. magnatum* fruiting bodies is positively related to the presence of mycelium in the soil although the fructification process is limited in space by other factors which are still not clear.

In previous studies of *T. melanosporum* it was found that the presence of a burnt area around a tree infected by *T. melanosporum* was related to the quantity of its mycelium in the soil [20]. These Authors, however, found a higher quantity of the mycelium in non-productive trees and explained this as a shift in resource allocation by the fungal ascoma. In our study we found the highest quantity of *T. magnatum* DNA in the productive plots, indicating that this truffle species has a different behaviour in the soil. As *T. magnatum* mycorrhizas are rare or absent in the productive areas and probably unable to support fruiting body formation, its free live mycelium should provide a sufficient quantity of nutrients to support ascoma formation and successive development. It has already been shown that after their formation in the soil truffle ascomata have a saprobic phase and, during their maturation, become nutritionally independent on the host plant. Probably, in *T. magnatum,* this saprobic phase is much more important than previously considered and as also suggested by Zampieri et al. [15].

## Conclusions

The results reported here demonstrate that the real-time PCR assay developed in this study can be an effective tool for quantifying *T. magnatum* in the soil and for monitoring the presence of this precious fungus, regardless of truffle production. This technique could be a useful tool to evaluate the “health” of natural and cultivated truffières and to assess the effect of different cultivation techniques. This aspect is particularly important because in natural truffières ascoma production is dispersed and depends on annual climatic conditions. Thus many years of survey are necessary to evaluate the effects of any new variable. Moreover, it is difficult to assess truffle production in natural truffières because in Italy there is no control of truffle harvesting in the forests and numerous different truffle hunters may visit a single truffière in one day [1].

Real-time PCR will make it possible to carry out further studies on the spatial and seasonal changes in the quantity of *T. magnatum* mycelium in the soil to gain more knowledge on its biology and ecology.

## Methods

### Experimental truffières

For this study four natural *T. magnatum* truffières located in four different Italian regions (Emilia Romagna, Tuscany, Abruzzo and Molise) were chosen on the basis of their high *T. magnatum* ascoma productivity. All these truffières are closed to the public so the scientific data on production collected are more meaningful.

The Emilia Romagna experimental truffière is located in the Museum of the Bonifica Renana park at Argenta (Ferrara) (latitude 44° 37′ 10″ N, longitude 11° 48′ 55″ E, altitude 5 m asl). This truffière is representative of the natural *T. magnatum* production areas in the Po valley that are mostly located in private or public gardens and parks, the natural indigenous forest having been largely supplanted by agriculture. The putative *T. magnatum* host plants are poplar (*Populus nigra* L.) and linden (*Tilia vulgaris* Hayne). The soil of the truffière is calcareous (10–25% of total CaCO_3_) with a pH ranging from 7.9 to 8.3 in the different plots.

The Tuscany, Abruzzo and Molise experimental truffières are representative of the natural *T. magnatum* truffières in the broad-leaved forests of the Apennine mountains of central-southern Italy. The Tuscan truffière is located at Barbialla nuova, Montaione (Florence) (latitude 43° 35′ 30″N, longitude 10° 50′ 55″ E, altitude 135 m asl). The putative host plants are hornbeam (*Ostrya carpinifolia* Scop*.*), poplar (*Populus alba* L.) and oaks (*Quercus cerris* L., *Quercus petraea* (Mattuschka) Liebl., *Quercus ilex* L.). The soil has a CaCO_3_ content ranging from 4 to 10% and a pH of 7.7-8.4.

The Abruzzo and Molise truffières are located in two Man & Biospher reserves managed by the Biodiversity Office of the State Forestry Corps: Feudozzo (Abruzzo) (latitude 41° 45′ 55″ N, longitude 14° 11′ 12″ E, altitude 950 m asl), and Collemeluccio (Molise) (latitude 41° 42′ 07″ N, longitude 14° 20′ 34″ E, altitude 810 m asl). In both areas there is a large contingent of meso-hygrophilous species, favoured by the presence of surface water, probably due to the proximity of small springs. There are many putative host plants in both truffières: at Feudozzo (Abruzzo) poplar (*Populus tremula* L.), oak (*Q. cerris*), willow (*Salix alba* L., *Salix apennina* Skvortsov, *Salix caprea* L. and *Salix purpurea* L.), hornbeam (*Carpinus betulus* L. and *Carpinus orientalis* Miller) and hazelnut (*Corylus avellana* L.); at Collemeluccio (Molise) poplar (*P. nigra* and *P. canadensis* L.), oak (*Q. cerris*), linden (*Tilia platyphyllos* Scop.), silver fir (*Abies alba* Miller), hazelnut (*C. avellana*) and hornbeam (*O. carpinifolia*). However, all *T. magnatum* collection occurred beneath *A. alba*. The geological substratum is represented by alternating argillaceous sandstone: at Feudozzo, the soil has a CaCO_3_ content ranging from 0.75 to 4.20% and a pH of 6.8-7.8; at Collemeluccio the soil has a CaCO_3_ content ranging from 1.69 to 2.64% and a pH of 6.8-7.4.

As production areas are often of different dimensions and their productivity varies considerably, in the experimental truffière productive plots of 300–500 m^2^ were selected on the basis of the confidential indications of their productivity provided by local truffle hunters and their real productivity was established over the three years of the study. A total of 39 plots (9 in Tuscany, 9 in Emilia Romagna, 9 in Molise and 12 in Abruzzo) were identified and delimited. Details of the pedological and vegetative characteristics of each experimental truffière plot are described in the project website [36-38].

### Assessment of truffle production

We used trained dogs to assess truffle production every week in the *T. magnatum* season (September-December) for three consecutive years (2008–2010). The truffles collected were numbered, weighed and recorded for each plot.

### Experimental layout

Soil cores (1.6 cm diameter, 30 cm deep) were extracted using a disposable, cylindrical, polyvinyl chloride tube inserted inside a steel soil borer, purpose-built for this study. A set of 9 equidistant soil cores were taken from each plot along two diagonal lines, excluding a border area of 5 m on each side of the plot to minimize possible edge effects. Sampling was carried out in January 2009, 2010 and 2011 at the end of the annual white truffle season.

The soil cores collected from each plot were pooled together to obtain a sample per plot for each year and any root fragments, stones or organic debris were carefully removed using a stereomicroscope. A control soil sample was also collected 200 m outside each experimental truffière from non-productive areas. The soil was stored at −80°C and then lyophilized the for three days using the Virtis Benchtop 2 K freeze dryer (SP Industries, Gardiner, New York). After drying, each sample was finely ground in a mortar, sieved, homogenized and stored at −20°C until DNA extraction was performed.

### Soil DNA extraction

A DNA extraction procedure was specifically developed for all the four types of soil analysed in this study. Three replicates (5 g each) were prepared for each soil sample, re-suspended in 6–7 ml of CTAB lysis buffer (2% CTAB, 2% Polyvinylpyrrolidon, 2 M NaCl, 20 mM EDTA, 100 mM Tris–HCl, pH 8) and processed according the detailed protocol described in Additional file 2. Brown crude DNA solutions (about 3 ml in volume) from each reaction were obtained following this extraction phase and 1 ml aliquots were then purified using the Nucleospin Plant II kit (Macherey-Nagel, Düren, Germany) following the manufacturer’s instructions with slight modifications (see Additional file 2). Total DNAs were finally eluted in 65 μl of elution buffer (5 mM Tris/HCl, pH 8.5). The amount of DNA in each extract was quantified using a NanoDrop ND-1000 Spectrophotometer (Thermo Scientific). The quality of the total DNAs was evaluated with optical density (OD) 260/280 nm and 260/230 nm ratios. Extractions with OD ratios less than 1.4 and DNA quantity less than 25 ng μl^–1^ were repeated. In addition soil DNA extracts were PCR-amplified with primer pair ITS1-ITS4 [39] to confirm the absence of DNA polymerase inhibitors. Extracts with positive ITS1-ITS4 amplification products (from 500 bp to 1000 bp) were considered suitable for quantitative PCR (qPCR) assays. Purified DNAs were stored at −80°C until processed.

### Primer and probe selection

ITS1-5.8 S-ITS2 rDNA sequences of *T. magnatum* and other truffle species were retrieved from GenBank database (http://www.ncbi.nlm.nih.gov/; date of accession: June, 2008) and aligned with Multalign [40] to identify species-specific domains for primer and probe selection. Oligonucleotide design was carried out with Primer3 software (http://frodo.wi.mit.edu/primer3/) [41] with the following parameters: amplicon size 90–110, primer size 18–22 bp (opt. 20 bp), melting temperature 58-62°C (opt. 60°C), GC content 40-60% (opt. 50%), Max Self Complementarity = 5. Secondary structures and dimer formation were verified using Oligo Analyzer 1.0.3 software (Freeware, Teemu Kuulasmaa, Finland) and specificity was firstly evaluated *in silico* using BLASTN algorithm (http://blast.ncbi.nlm.nih.gov/Blast.cgi). A primer pair and the respective probe was selected for both the ITS1 and the ITS2 region (Table 2) and their specificity was then confirmed with qualitative PCR against genomic DNA of different mycorrhizal, saprobic and pathogenic fungi (Table 3). The specificity of the oligonucleotides selected as probes was tested in PCR reactions using their opposite primers (TmgITS1rev with TmgITS1prob and TmgITS2for with TmgITS2prob). Fungal DNA was isolated from fruiting bodies or mycelia using the Nucleospin Plant II kit (Macherey-Nagel) according to the manufacturer’s protocol for fungi. Furthermore, the sensitivity of the selected primer pairs was assessed by amplifying *T. magnatum* DNA 10-fold serial dilutions (from 10 ng to 0.001 ng) in pooled genomic DNAs from the other fungal species used in this study.

Conventional PCRs were performed on 25 μl reaction mixture volumes containing 100 ng of total DNA, 10 mM Tris–HCl (pH 8.3), 50 mM KCl, 1.5 mM MgCl_2_, 200 μM for each dNTP, 400 nM for each primer and 1.5 U of TaKaRa^TM^ rTaq DNA polymerase (Takara, Otsu, Japan). PCR conditions were as follow: 25 cycles of 95°C for 20 s, 60°C for 30 s, 72°C for 40 s with an initial denaturation at 95°C for 6 min and a final extension at 72°C for 7 min. PCR products were electrophoresed in 1% agarose gels and visualized by staining with ethidium bromide in a GeneGenius Imaging System (SynGene, Cambridge, UK).

### Real-time PCR

TaqMan PCR assays were carried out in 96-well optical plates (Bioplastic) using a Stratagene Mx3000P QPCR system (Stratagene, La Jolla, CA, USA). Each amplification was performed on 25-μl reaction volumes containing 12.5 (1X) μl of Maxima Probe qPCR Master mix (Fermentas), 30 nM of ROX and 200 ng of total DNA. Primer and probe concentration were optimised to 0.5 μM and 0.2 μM respectively based on the lowest threshold cycle (C_t_) values and the highest fluorescent signal. The TaqMan probe was labelled at the 5’end with the fluorescent reporter dye FAM (6-carboxy-fluorescin) while the 3′ end was modified with the quencher dye TAMRA (6-carboxy-tetramethylrhodamine) (MWG BIOTECH, Ebersberg, Germany). Two replicates per soil sample and no template controls were prepared for each plate and Real-time PCRs were repeated twice to confirm the results.

The optimised thermal cycle protocol included a 10 min incubation at 95°C followed by 45 cycles of 95°C for 15 s, 60°C for 30 s and 72°C for 30 s. The threshold fluorescence level was determined with the default adaptive baseline algorithm of the MXPro software (version 4.10) (Agilent technologies) and the resulting C_t_ values were automatically converted to quantities of *T. magnatum* DNA using the standard curve method. A standard curve was generated for each run with a series of ten-fold dilutions of genomic DNA from *T. magnatum* (from 10^7^ to 10^2^ fg per reaction) as standards. To evaluate the real-time PCR detection limit further serial dilutions of 1 and 10 fg of *T. magnatum* DNA were tested in triplicate. All real-time PCR products were electrophoresed as described above to exclude amplification of non-target sequences.

### Data analysis

ANOVA was applied to check for significant differences in the amount of DNA extracted and the *T. magnatum* DNA concentrations obtained from the different trufféres. When significant differences were encountered, mean values were compared using Bonferroni’s test. The non**-**parametric Kruskal**-**Wallis test was used to verify the results obtained with the ANOVA. Spearman’s rank correlation coefficient was calculated to determine correlations between *T. magnatum* DNA concentration and truffle production (ascoma number and weight). The significance level was set at the 5% probability level. Statistical analyses were performed using XLSTAT- Pro 7.5 (Addinsoft, Paris, France).

## Abbreviation

OD, Optical density; ITS, Internal transcribed spacer; Asl, Above sea level; CTAB, Cetyl Trimethyl Ammonium Bromide; EDTA, Ethylenediaminetetraacetic acid disodium salt; Ct, Threshold cycle; FAM, 6-carboxy-fluorescin; TAMRA, 6-carboxy-tetramethylrhodamine.

## Competing interests

The authors declare that they have no competing interests.

## Authors’ contributions

MI participated in the design of the study, perfected the DNA extraction method, processed and analysed Emilia Romagna and Tuscany samples, performed Real Time analyses and helped to draft the manuscript. ML contributed in coordination of the study and helped in processing Molise and Abruzzo samples. MO processed and analysed Molise and Abruzzo samples. ES participated in processing Tuscany samples and carried out the statistical analyses. EB helped to perform Real Time analyses and to analyse the data. AZ participated in the study conception and coordination and drafted the manuscript. All authors read and approved the final version of the manuscript.

## Supplementary Material

Additional file 1 **Number and weight of ascomata.** This file contains a table showing the number and weight of the ascomata found in the experimental plots of the four truffières over the three years of survey (2008-2009-2010). Click here for file

Additional file 2: **DNA extraction protocol.** This file contains the detailed protocol developed in this study for the extraction of genomic DNAs from 5 g soil samples. Click here for file
