# Supplementary material for: Development and validation of a real-time PCR assay for detection and quantification of Tuber magnatum in soil
Source: BMC Microbiol. 2012 Jun 6;12:93. doi: 10.1186/1471-2180-12-93 (PMC3438110; doi:10.1186/1471-2180-12-93)
Supplement: Additional file 1 — Number and weight of ascomata. This file contains a table showing the number and weight of the ascomata found in the experimental plots of the four truffières over the three years of survey (2008-2009-2010). [file 1471-2180-12-93-S1.doc]

**Supplemental Table 1 Number and weight of the ascomata found in the experimental plots during the three years of survey.**

| Plot^1^ | year | | | | | | total | |
| --- | --- | --- | --- | --- | --- | --- | --- | --- |
|  | 2008 | | 2009 | | 2010 | |  |  |
|  | n. | g | n. | g | n. | g | n. | g |
| Fe-1 | 0 | 0 | 1 | 11 | 0 | 0 | 1 | 11 |
| Fe-2 | 0 | 0 | 1 | 8 | 2 | 21.4 | 3 | 29.4 |
| Fe-3 | 0 | 0 | 0 | 0 | 0 | 0 | 0 | 0 |
| Fe-4 | 0 | 0 | 0 | 0 | 0 | 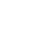0 | 0 | 0 |
| Fe-5 | 2 | 5.7 | 2 | 21.5 | 6 | 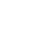100 | 10 | 127.2 |
| Fe-6 | 0 | 0 | 0 | 0 | 0 | 0 | 0 | 0 |
| Fe-7 | 0 | 0 | 0 | 0 | 0 | 0 | 0 | 0 |
| Fe-8 | 0 | 0 | 0 | 0 | 0 | 0 | 0 | 0 |
| Fe-9 | 0 | 0 | 0 | 0 | 3 | 30 | 3 | 30 |
| Fe-10 | 0 | 0 | 0 | 0 | 0 | 0 | 0 | 0 |
| Fe-11 | 0 | 0 | 5 | 75 | 0 | 0 | 5 | 75 |
| Fe-12 | 0 | 0 | 2 | 55 | 0 | 0 | 2 | 55 |
| Ar-1 | 0 | 0 | 0 | 0 | 0 | 0 | 0 | 0 |
| Ar-2 | 1 | 4 | 1 | 20 | 0 | 0 | 2 | 24 |
| Ar-3 | 0 | 0 | 0 | 0 | 0 | 0 | 0 | 0 |
| Ar-4 | 0 | 0 | 0 | 0 | 4 | 50 | 4 | 50 |
| Ar-5 | 0 | 0 | 0 | 0 | 0 | 0 | 0 | 0 |
| Ar-6 | 5 | 118 | 1 | 10 | 1 | 20 | 7 | 148 |
| Ar-7 | 2 | 40 | 0 | 0 | 0 | 0 | 2 | 40 |
| Ar-8 | 0 | 0 | 0 | 0 | 0 | 0 | 0 | 0 |
| Ar-9 | 0 | 0 | 0 | 0 | 0 | 0 | 0 | 0 |
| Ba-1 | 0 | 0 | 0 | 0 | 0 | 0 | 0 | 0 |
| Ba-2 | 3 | 24 | 0 | 0 | 0 | 0 | 3 | 24 |
| Ba-3 | 0 | 0 | 0 | 0 | 2 | 14 | 2 | 14 |
| Ba-4 | 1 | 10 | 1 | 8 | 1 | 8 | 3 | 26 |
| Ba-5 | 2 | 48 | 0 | 0 | 1 | 80 | 3 | 128 |
| Ba-6 | 0 | 0 | 0 | 0 | 0 | 0 | 0 | 0 |
| Ba-7 | 2 | 15 | 0 | 0 | 1 | 18 | 3 | 33 |
| Ba-8 | 2 | 80 | 2 | 17 | 0 | 0 | 4 | 97 |
| Ba-9 | 0 | 0 | 0 | 0 | 0 | 0 | 0 | 0 |
| Co-1 | 0 | 0 | 0 | 0 | 0 | 0 | 0 | 0 |
| Co-2 | 0 | 0 | 0 | 0 | 0 | 0 | 0 | 0 |
| Co-3 | 0 | 0 | 0 | 0 | 0 | 0 | 0 | 0 |
| Co-4 | 0 | 0 | 0 | 0 | 0 | 0 | 0 | 0 |
| Co-5 | 0 | 0 | 0 | 0 | 0 | 0 | 0 | 0 |
| Co-6 | 0 | 0 | 0 | 0 | 0 | 0 | 0 | 0 |
| Co-7 | 0 | 0 | 0 | 0 | 0 | 0 | 0 | 0 |
| Co-8 | 2 | 28 | 9 | 139 | 6 | 105.7 | 17 | 272.7 |
| Co-9 | 0 | 0 | 0 | 0 | 0 | 0 | 0 | 0 |

^1^The fist two letter indicate the locality (Fe = Feudozzo, Abruzzo; Ar = Argenta, Emilia Romagna; Ba = Barbialla, Tuscany; Co= Collemeluccio, Molise), and the last number the plot number.
