# Supplementary material for: Development and validation of a real-time PCR assay for detection and quantification of Tuber magnatum in soil
Source: BMC Microbiol. 2012 Jun 6;12:93. doi: 10.1186/1471-2180-12-93 (PMC3438110; doi:10.1186/1471-2180-12-93)
Supplement: Additional file 2: — DNA extraction protocol. This file contains the detailed protocol developed in this study for the extraction of genomic DNAs from 5 g soil samples. [file 1471-2180-12-93-S2.doc]

**Detailed protocols for DNA extraction from soil**

**CTAB lysis buffer (**pH 8)**:**

2M NaCl

20 mM EDTA

100 mM Tris-HCl

2% (w/v) CTAB

2% (w/v) Polyvinylpyrrolidon (MW 25000)

1. Weight 5 g of freeze-dried powdered soil in a 15 ml-polypropylene tube and add 1 g of acid-washed glass beads (150-212 μm)
2. Add 6-7 ml of the CTAB lysis buffer and vortex vigorously until the soil is completely soaked
3. Heat the soil-containing tube in a microwave oven (450 W) for 8 s
4. Add 100 µl of a lysozyme solution (100 mg/ml)
5. Shake the tube horizontally (3000 rpm) for 10 min on a vortex provided with platform
6. Incubate the tube at 37 °C for 30 min
7. Add 300 µl of a SDS solution (200 mg/ml) and vortex vigorously
8. Incubate the tube for 30 min at 65ºC in a water bath and mix the contents by inversion every 5 min
9. Centrifuge the samples at 5000 g for 10 min at room temperature
10. Transfer the upper phase to a new 15 ml-polypropylene tube
11. Add 1 volume of phenol-chloroform-isoamyl alcohol (25:24:1) and vortex for 10 s
12. Centrifuge the samples at 5000 g for 1 min at room temperature
13. Transfer 1 ml of the upper phase (~ 3 ml in total) to a new1.5 ml-microcentrifuge tube

The crude DNA solutions (1 ml for each sample) were then purified by NucleoSpin Plant II (Macherey-Nagel) according the manufacturing instructions with slight modifications. DNA washing steps with the ethanol-containing buffer (PW2) were increased from 2 to 3, using 700 μl, 400 μl and 200 μl respectively. Final elution was carried out with 65 μl of 5 mM Tris/HCl buffer (pH 8.5)
